# Tissue-Specific Decellularized Extracellular Matrix Bioinks for Musculoskeletal Tissue Regeneration and Modeling Using 3D Bioprinting Technology

**DOI:** 10.3390/ijms22157837

**Published:** 2021-07-22

**Authors:** Wonbin Park, Ge Gao, Dong-Woo Cho

**Affiliations:** 1 Department of Mechanical Engineering, Pohang University of Science and Technology, Pohang 37673, Korea; wbpark@postech.ac.kr; 2 Institute of Engineering Medicine, Beijing Institute of Technology, Beijing 100081, China; gaoge@bit.edu.cn; 3 POSTECH-Catholic Biomedical Engineering Institute, Pohang University of Science and Technology, Pohang 37673, Korea; 4 Institute of Convergence Science, Yonsei University, 50 Yonsei-ro, Seodaemun-gu, Seoul 03722, Korea

**Keywords:** musculoskeletal tissue, tissue engineering, 3D bioprinting, decellularized extracellular matrix bioink

## Abstract

The musculoskeletal system is a vital body system that protects internal organs, supports locomotion, and maintains homeostatic function. Unfortunately, musculoskeletal disorders are the leading cause of disability worldwide. Although implant surgeries using autografts, allografts, and xenografts have been conducted, several adverse effects, including donor site morbidity and immunoreaction, exist. To overcome these limitations, various biomedical engineering approaches have been proposed based on an understanding of the complexity of human musculoskeletal tissue. In this review, the leading edge of musculoskeletal tissue engineering using 3D bioprinting technology and musculoskeletal tissue-derived decellularized extracellular matrix bioink is described. In particular, studies on in vivo regeneration and in vitro modeling of musculoskeletal tissue have been focused on. Lastly, the current breakthroughs, limitations, and future perspectives are described.

## 1. Introduction

The musculoskeletal system is a major body system comprising bones, cartilages, muscles, tendons, ligaments, and other connective tissues. It protects internal organs from external danger, supports locomotion including stability and mobility, and provides other homeostatic functions such as storage of minerals and hematopoiesis [1]. Unfortunately, musculoskeletal disorders are the leading cause of disability worldwide, and over 1.7 billion people suffer from functional impairment of the system [2]. Despite its regenerative capacity against small damage, the locomotor system is incapable of fully regenerating its functions for large-volume loss and demands interventional support [3]. Conventionally, autografts, allografts, and xenografts have been implanted into defective sites; however, there are several drawbacks, including a shortage of donor organs and immune rejection [4]. Therefore, to overcome these limitations, innovative tissue engineering approaches have been proposed based on an understanding of the complexity of human musculoskeletal tissues.

**Figure 1:**
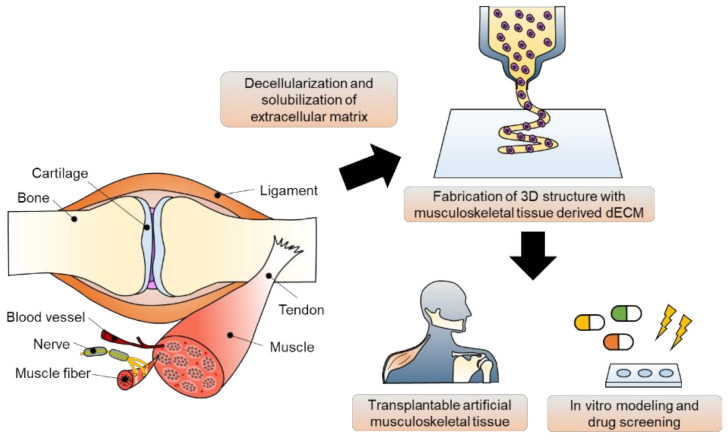
Musculoskeletal tissue derived decellularized extracellular matrix (dECM) bioink preparation and its applications in 3D bioprinting-based musculoskeletal tissue engineering.

Tissue engineering strategies have been applied for the in vivo regeneration and in vitro modeling of musculoskeletal tissue (Figure 1). These strategies can be classified as scaffold-based and scaffold-free approaches. The scaffold is defined as a guiding frame-work for cell functions, including adhesion, proliferation, and differentiation [5]. Using various technologies, such as molding, electrospinning, soft lithography, and micropatterning, scaffolds made of polydimethylsiloxane, poly(2-hydroxyethyl methacrylate), elastin, collagen, and fibrin have been fabricated for musculoskeletal tissue engineering [6,7,8,9,10,11]. Unlike the classical tissue engineering strategy, scaffold-free tissue equivalents provide a biomimetic microenvironment supporting cellular communication. Based on cell-sheet engineering and pellet/aggregate/spheroid culture platforms, the physiological and functional features of musculoskeletal tissue have been recapitulated in vitro [12,13]. Studies on vascularization and innervation in musculoskeletal tissue have also been reported using scaffold-free implants [14]. By stimulating the innate regenerative capacity of native tissue, tissue-engineered constructs have shown substitutional potential. However, recapitulating the three-dimensional organ structure and complex heterogeneity of the tissue microenvironment using conventional tissue engineering approaches is difficult because of the intrinsic inability to precisely define the spatial distribution of cells [15].

Amongst many tremendous advances, 3D bioprinting technology has been regarded as one of the most promising technologies that can overcome the weaknesses of traditional tissue engineering approaches, leading to technological revolution in the tissue engineering field. By depositing biochemical components to the desired site, the structural and physiological heterogeneity of the organ microenvironment can be recapitulated [16,17]. By reproducing the physiological complexities of human tissue, including the diversity of cells, the surrounding structural proteins, and soluble cytokines, in three-dimensional structures, a highly developed approach for tissue regeneration has become possible [18,19]. In addition to the regenerative approach, in vitro modeling for physiological and pathological studies of musculoskeletal tissue has also come into greater prominence recently. The 3D-bioprinted in vitro platforms, such as spheroids, organoids, and organ-on-chips containing multiple components, would benefit tissue modeling through drug discovery and the development of precise medicine and patient-specific regenerative therapies [20,21].

Bioink, a mixture of biocompatible hydrogels and functional cells, is a key element in 3D bioprinting [22]. Numerous bioinks, including synthetic polymers and natural hydrogels, have been investigated to replicate the native tissue microenvironment to develop an effective tissue engineering strategy [23]. Among them, decellularized extracellular matrix (dECM) has been spotlighted as one of the best therapeutic materials available because it inherits tissue- and organ-specific biochemical and physiological characteristics. In addition to basement membrane structural macromolecules, various functional components, such as growth factors and cytokines, can also be provided [22]. Many studies have shown that dECM bioinks applied to multiple areas, including the brain, heart, liver, pancreas, cornea, and skin, exert synergistic effects on cell survival, growth, migration, and differentiation [24,25,26,27,28,29,30]. Recently, the importance of musculoskeletal tissue engineering has been magnified, which has triggered the active development of musculoskeletal tissue-derived dECM bioinks [31,32].

This review aims to introduce the recent advances and accomplishments in musculoskeletal tissue engineering. Together with a description of the features of 3D bioprinting technology and bioprintable materials, effective production strategies for dECM biomaterials and key considerations for grafting them as 3D bioprintable bioinks are explained. The applications of 3D-bioprinted musculoskeletal equivalents using tissue-specific bioinks for both tissue repair and in vitro modeling will be the focus of this review. Lastly, recent advances, limitations, and future perspectives are discussed.

## 2. Three-Dimensional Bioprinting Technology and Bioinks

### 2.1. A Brief Overview of 3D Bioprinting Technology

Three-dimensional bioprinting has emerged as a revolutionary technology for building tissue-engineered constructs using datasets acquired by medical imaging devices, and has been widely applied in tissue regeneration, pathology studies, drug screening, and medical imaging. By depositing multiple biomaterials and living cells layer-by-layer, 3D bioprinting can precisely define 3D complex constructs that structurally emulate natural tissues/organs. Due to these advantages and demands, several 3D bioprinting companies, such as EnvisionTEC (Detroit, MI, USA), REGENHU (Villaz-St-Pierre, Switzerland), Poetis (Pessac, France), Organovo (San Diego, CA, USA), and nScrypt (Orlando, FL, USA), have commercialized various 3D bioprinters; further technological development is ongoing [33]. According to the working principle, 3D bioprinting technology can be classified into three sub-types: inkjet-based, laser-assisted, and microextrusion-based printing. Each type has distinct advantages and limitations. Therefore, to select an appropriate module for fabricating a tissue/organ construct with specific characteristics, it is important to understand their working mechanisms.

#### 2.1.1. Inkjet-Based 3D Bioprinting

**Figure 2:**
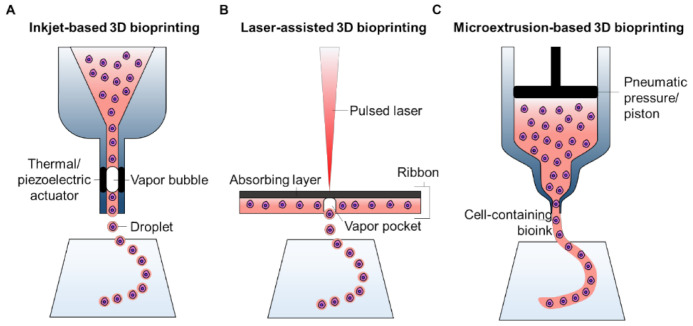
Schematic illustration of 3D bioprinting systems, including (**A**) inkjet-based, (**B**) laser-assisted, and (**C**) microextrusion-based 3D bioprinting technology.

Similar to traditional 2D printing, which ejects a tiny amount of ink onto paper to obtain an edited document, inkjet-based 3D bioprinting deposits nano- or micro-liter droplets of cell-laden bioinks toward a substrate in a drop-on-demand manner to stack-up a three-dimensional objective [34]. In general, the bioink containing living cells is loaded in a cartridge connected to the printing heads of an inkjet printer (Figure 2A). During the printing process, printing heads are usually deformed by a piezoelectric actuator or by thermal variation to squeeze the bioink, thus creating droplets. The piezoelectric heads include an element composed of special materials (e.g., crystals and ceramics) that are sensitive to electrical charges. As a pulsed voltage is provided, the elements bend backward, forcing an accurate amount of bioink from the cartridge onto the substrates [35]. A thermal inkjet printer imposes a rapid increase in temperature near the emission tip to generate a vapor bubble that outputs a droplet of bioink [36]. Recently, electrostatic and electrohydrodynamic-based modules have been adopted for 3D inkjet bioprinting techniques, which can achieve an identical effect by intermittently applying pressure to the bioink cartridge [37]. Several important parameters of inkjet-based 3D bioprinting must be considered to control and stabilize the droplets. The size of the droplets depends on the viscosity and surface tension of the bioink. The vibrational frequency of electrical signals that activate the actuators mainly dominates the speed of droplet deposition; the pulse and amplitude determine the consistency of the droplet number and size during each ejection.

Because of its ability to print small volumes of bioink, inkjet-based bioprinting has a high resolution, which is as fine as 2 μm, i.e., the dimension of a single cell [38]. Moreover, the rapid and tunable printing speed (1–250,000 droplets per second) is another merit of this technology [39]. However, the working mechanism requires careful selection of bioink with low viscosity (<0.1 Pa·s) to prevent nozzle clotting [40], which drastically limits the cell density upper range and narrows the range of available biomaterials.

#### 2.1.2. Laser-Assisted 3D Bioprinting

Comparable with inkjet printing, laser-assisted printing also generates cell-laden droplets using a laser beam and deposits them on a substrate to build a 3D construct. A typical laser-assisted bioprinting system consists of three elements: a light source that focuses a pulsed laser beam; a ribbon composed of a laser-absorbing layer sandwiched between a transparent donor slide and a layer of cell-laden biomaterial; and a receiving reservoir or substrate that facilitates droplet deposition and cell adhesion [41]. During the printing process, as the laser beam is focused on the ribbon, the materials in the absorbance layer (e.g., Au, Ti) produce vapor pockets that induce the formation of droplets and propel them onto the substrate (Figure 2B). The key parameters to manipulate a laser-assisted bioprinter include laser effluence (e.g., laser wavelength, pulse energy), elimination frequency, the surface tension of the substrate, the distance between the ribbon and substrate, and the thickness and viscosity of the cell-laden layer.

Laser-assisted bioprinting has the advantages of ultrafine resolution (20–30 μm) and rapid printing speed (5 kHz) [42]. In addition, because of the nozzle-less working mechanism, this technology is not subjective to the cell density and viscosity of biomaterials. Despite these advantages, owing to the sophisticated laser system and composite ribbon, the costs of establishing a laser-assisted bioprinting system are inevitably high, which limits the widespread adoption of this technique.

#### 2.1.3. Microextrusion-Based 3D Bioprinting

Driven by a pneumatic or mechanical system, microextrusion-based bioprinting continuously forces cell-laden bioinks through a nozzle, forming a fine filament. By controlling the movement of the nozzles, the extruded filaments are stacked to create 3D constructs (Figure 2C). A pneumatic system applies stable air pressure provided by a clean compressed air source to the bioinks residing in a cartridge. Because the pressure values can be flexibly manipulated, a wide range of biomaterials with various viscosities (30 mPa·s to over 6 × 10^7^ mPa·s) can be fabricated for bioprinting tissue/organ equivalents [43]. In comparison, the mechanical system directly imposes forces on the bioinks via a piston or screw module. While the steady movement of the piston module is beneficial for tuning the flow rate of the bioink, the screw module offers better spatial control on the cartridge, thereby extruding the bioinks with higher viscosity [44]. However, the severe mechanical force might have detrimental effects on the cells encapsulated in the bioink owing to the potential damage to the cell membrane, leading to cell death.

Although microextrusion-based bioprinting has a broader pool of applicable biomaterials, the printing resolution, in comparison with the other two aforementioned technologies, is inevitably lower (>100 μm) owing to the shear stress [45]. During the extrusion process, the shear stress can exceed a threshold that secures the viability of the loaded cells when the nozzle size is small and the viscosity of the bioink is high. A study has reported that highly sensitive cells retain high cell viability (≥90%) after printing as long as a low shear stress (~100 Pa) is ensured [46]. Therefore, to fabricate a highly viable tissue construct, it is important to increase the nozzle size and reduce the viscosity of the bioink, which may result in a decreased printing resolution. However, the relatively larger nozzle used in microextrusion-based bioprinting systems can lead to a high printing speed, enabling rapid fabrication of large-volume constructs. Moreover, the rough resolution allows for direct bioprinting of pre-processed cell spheroids/aggregates, which could rapidly create functional tissues via cell self-assembly.

In summary, the important considerations and performances of three different 3D bioprinting systems are compared in Table 1. In general, each bioprinting technique has its advantages and limitations. Despite the relatively low resolution, microextrusion-based bioprinting offers a solution for the rapid fabrication of volumetric constructs using viscose bioinks with high cell density. Owing to these advantages, among bioprinting technologies, the microextrusion-based approach is the most promising technology for building tissue/organ substitutes thus far. Several pioneering reports have successfully created human-sized ears, muscles, tendons, and hearts.

### 2.2. Bioinks for 3D Bioprinting of Living Constructs

Various biomaterials, such as biocompatible metals, ceramics, bio-glasses, and polymers, have demonstrated their superiority when engineered as scaffolds for tissue repair and regeneration. Most of these materials are also applicable to traditional 3D printing techniques (e.g., selective laser sintering, fused deposition modeling, and stereolithography) [47]. However, for 3D bioprinting of living cells, the bioink should protect the viability and activity of the encapsulated cells. One fundamental requirement is to provide a humid and amicable environment. Hence, cell-friendly polymers that contain a high water content, such as hydrogels, are the prevailing options for bioinks, narrowing the range of biomaterials applicable for 3D bioprinting. In addition to escorting living cells, the bioink also plays an important role in facilitating the fabrication process. Therefore, acceptable printability is another necessary performance requirement for a bioink; this refers to the appropriate rheological properties (e.g., suitable viscosity and shear-thinning behavior) and the capacity for sol-gel transition. In addition, to maintain the printed structures and emulate the mechanical features of various natural tissues, bioinks that exhibit tunable mechanical properties (e.g., strength and modulus) after polymerization are desired.

In general, polymers widely used in 3D bioprinting technology can be divided into two groups: synthetic polymers (e.g., polyethylene glycol, Pluronic F-127, poly(N-isopropylacrylamide)) and naturally derived polymers (e.g., collagen, gelatin, fibrin, silk fibroin, hyaluronic acid (HA), alginate, chitosan, and decellularized extracellular matrix (dECM)). Synthetic polymers contain macromolecules that are artificially produced through the synthesis of primary materials from gas, oil, or coal. By changing the molecular weight and controlling the chemical polymerization, the rheological and mechanical properties of synthetic polymers can be readily tuned [48]. Such mechanical flexibility has provoked numerous attempts to develop strong and robust cell-laden hydrogels to emulate the mechanical properties of native load-bearing tissues. However, these materials generally lack cell-adhesive ligands, resulting in a limited ability to support cells activities, thus limiting their medical applications [49]. The recapitulation of cell-friendly physiological functions beyond structural replication is the main core of 3D bioprinting-based tissue engineering.

In contrast, naturally derived polymers usually originate from various natural sources (e.g., plants, animals, and microorganisms) and are composed of natural substances such as proteins, polysaccharides, and their derivatives [50]. The solubilized extracellular matrix (ECM) molecules can only be gathered via unstable bonding forces such as hydrogen bonds, ionic bonds, and weak covalent bonds, rather than being tightly assembled to form strong fibers as are present in native bodies. Thus, bioinks developed from natural polymers are usually fragile and show poor printability. Despite this drawback, the irresistible merit of natural polymers is their high similarity to native ECM compositions and structures, which can offer a favorable microenvironment to promote the activities of encapsulated cells (e.g., adhesion, migration, proliferation, and differentiation) and thus benefit the morphogenesis of functional tissues [51], as summarized in Table 2, which compares natural polymers with representative synthetic polymeric bioinks.

Among natural polymers, tissue-specific dECMs have particularly distinguishable characteristics. This type of material is directly obtained from native tissues by thoroughly removing the residing cells to minimize immunogenicity, leaving abundant natural ECM components [52]. dECM can inherit the unique functional components, compositional distributions, and spatial structures of diverse types of tissues, thereby providing a tissue-specific microenvironment to local cells. Such a “ready-to-live” niche is essential to encourage cells to exert their functions (e.g., population, aggregation, ECM deposition) to generate neo-tissues. Owing to this advantage, tissue-specific dECMs are considered as one of the most promising bioink sources for 3D bioprinting and have been applied to construct various tissues and organs (e.g., cardiac, muscle, adipose, cartilage, cornea, skin, blood vessels, and liver) [53].

In the remaining sections of this review, we describe the general approaches of decellularization and dECM bioink formulation, discuss the essential considerations of dECM bioink in the 3D bioprinting process, summarize their applications in constructing musculoskeletal tissue analogs for tissue regeneration and in vitro modeling, and conclude with some perspectives on the future and challenges of dECM-based bioinks.

## 3. Preparation of dECM Bioinks

### 3.1. General Methods for Producing dECM Bioink

The extracellular matrix (ECM) plays a crucial role in supporting cellular functionality and reinforcing tissue-specific phenotypes. Various ECM-derived materials, including collagen, gelatin, and HA, have been employed for in vivo and in vitro modeling. However, these materials have compositional limitations in recapitulating the microenvironment of tissues [54]. As an alternative strategy, the use of dECM has been proposed by many researchers. The tissue-specific composition of ECM proteins provides a compositional similarity with native tissue and their inherent biochemical and biophysical cues to promote cellular functions, including morphogenesis and development [55]. Furthermore, when combined with 3D printing technology—a cutting-edge technology—dECM undergoes complete transfiguration into bioink, encapsulating the cells and improving the degree of freedom for tissue engineering [56].

#### 3.1.1. Decellularization

Decellularization is a process that removes cellular and genetic molecules and preserves the acellular ECM components to maintain the mechanical and biochemical properties of tissue-specific microenvironments for artificial tissue engineering. The process can be classified into four main treatment categories: physical, chemical, enzymatic, and combinatorial [53]. Physical decellularization, including thermal, mechanical, and electrical treatments, has been reported in some studies. The main purpose of these physical approaches is to destroy the cellular and plasma membranes and remove biomolecules from the tissue. Thermal shock methods, including rapid freezing and snap freezing, are frequently employed. The growth of ice crystals weakens the cellular membrane and facilitates the release of cellular lysates. Direct abrasion with mechanical force through sonication and agitation accelerates cell-ECM dissociation [57]. However, physical methods should be followed by post-processing, coupled with chemical and enzymatic treatments to remove the membranous and intracellular lysates by promoting the diffusion of decellularizing agents [58].

Chemicals accelerate decellularization by decomposing chemical junctions, lysing cellular membranes, and removing cytoplasmic components. Diverse chemical decellularization reagents, including acids and alkalis, hypo/hypertonic solutions, ionic/non-ionic/bipolar detergents, and enzymes, have been employed [59]. Acids and alkalis, such as peracetic acid, hydrochloric acid, sodium hydroxide, and ammonium hydroxide, hydrolyze cytoplasmic biomolecules, including nucleic acids, and sterilize materials by neutralizing microbiomes. However, these pH-shifting solutions affect the ECM composition and lead to structural/functional changes [60,61]. Treatments with ionic/non-ionic/bipolar detergents induce ionic disturbance, resulting in dissociation of the cellular membrane and cytoplasmic proteins. Ionic detergents, such as SDS, have either a cationic or negatively charged head and are usually used to solubilize cellular and cytoplasmic membranes. However, due to their high destructivity, essential ECM components, including glycosaminoglycans (GAGs), collagen, and growth factors, can be eliminated [62]. Although nonionic detergents, including Triton X-100, are effective for delipidation, they have relatively weak detrimental effects that require long-term treatment, which may be an effective decellularization method to preserve biochemical and mechanical properties [63]. Bipolar detergents such as CHAPS, SB-10, and SB-16 have a neutral net charge and possess moderate disruptive capacity between that of ionic and nonionic detergents, which are appropriate characteristics for removing ECM proteins and DNA [64]. Hypotonic (e.g., Tris-HCl) and hypertonic (e.g., NaCl) solutions break down the matrix molecules and dissociate the junction between DNA and protein via an osmotic effect. Chelating agents, including EGTA and EDTA, bind to divalent cations, including Ca^2+^ and Mg^2+^, which mediate cell-ECM adhesion and disturb the protein–protein interactions, coupled with other enzymes and detergents [59].

Enzymes, including proteases, esterases, and nucleases, cleave intercellular and extracellular junctions as well as intracellular interactions. Proteolytic enzymes, including trypsin and dispase, cleave peptide bonds with high specificity. In particular, trypsin cuts the peptide bond between lysine and arginine and disrupts the ultrastructure of the native tissue in a time-dependent manner, preserving GAGs and breaking collagen and elastin, which changes their mechanical properties. Dispase cleaves basement membrane proteins, including collagen type IV and fibronectin. α-Galactosidase dissects alpha-galactosyl moieties of glycoproteins and glycolipids and prevents adverse xenoimmune responses. Nucleases, including endonucleases and exonucleases, cleave nucleic acids, including ribonucleotides and deoxynucleotides [65]. However, long-term enzymatic treatment drastically changes the mechanical and biochemical properties of the ECM. Furthermore, after enzymatic treatment, the lysate, including undesired ECM residues, needs to be removed through further processes to prevent an immune response.

As the terminal step of decellularization, sterilization is a critical process which removes the remaining pathogenic compounds and prevents an adverse immune response prior to in vitro analysis or implantation. Several sterilization methods can be employed, including dry heat, pressurized steaming, electron beam irradiation, gamma ray irradiation, and chemical treatments using ethylene oxide and peracetic acid. Because the biochemical and physical properties of dECM can be affected by disinfection, selecting the proper sterilization method is very important [66]. In addition to sterility, the effects on the deformation of growth factors and cytokines and the preservability of structural stability and biocompatibility should be satisfied.

#### 3.1.2. Compositional Quantification of dECM

The content of the prepared dECM should be quantified and qualified. The goals of decellularization are maximal clearance of cellular and genetic molecules and minimal loss of ECM components. Hematoxylin and eosin staining and DNA quantification are commonly conducted to verify the absence of cells and cell debris in the dECM. Using hematoxylin and eosin staining, the efficiency of decellularization can be determined by visualizing the presence of nuclei in the cells. Because double-stranded DNA (dsDNA) induces an immune response, the content of dsDNA should be quantified and should be lower than 50 ng dsDNA per mg dECM. Additionally, xenogenic factors such as the Gal epitope should be eliminated to prevent adverse inflammatory reactions in the recipient [67].

The preservation of ECM components, such as collagen, GAGs, and elastin, should also be estimated. Decellularization changes the composition of the extracellular matrix. In particular, the loss of components related to cellular functionality in the ECM influences tissue-specific cellular phenotypes due to the lack of attachment sites, growth factors, and other molecules. For instance, in the case of bone tissues, the content of collagen and hydroxyapatite should be precisely quantified because the components provide flexibility and strength for load-bearing functions. Recently, proteomic analysis has been highlighted because of its complementary strategy, providing both compositional and quantitative data. It also allows an in-depth study of enriched proteins such as growth factors and cytokines in dECM [68]. In addition, as well as the development of analysis tools, the process of decellularization should be also optimized in order to qualify the dECM components.

#### 3.1.3. Solubilization of dECM

Conventionally, dECM-based materials have been used in the form of whole organs or cell sheets for in vitro modeling. However, several limitations for tissue engineering, such as dimensional differences and a lack of tailored microgeometries, have been raised. To overcome these drawbacks, the soluble form of dECM can be utilized along with the development of 3D bioprinting technology [56]. A gel-like substance with the desired concentration is essential for constructing tissue analogs. Preserving tissue-specific biochemical properties, including essential growth factors and peptides, is important because each tissue has different cells that secrete different functional factors. Furthermore, the rheological properties need to be fulfilled for use as a bioink for 3D printing. This material is regarded as a new class of biomimetic hydrogels.

Solubilization is usually accomplished using enzymatic acidic digestion of the dECM to be printed. Briefly, small pieces of lyophilized dECM are digested using pepsin, an endopeptidase that catalyzes protein hydrolysis at pH 2.0–3.5. Together with physical stirring, a gel-like substance with the desired concentration can be obtained [69]. After digestion, the hydrogel is neutralized at a proper temperature to recover and spontaneously reform intramolecular bonds of the solubilized dECM protein [58]. Through this process, various dECM bioinks have been developed; several bioprinting companies, such as T&R Biofab and Innoregen, have recently successfully commercialized dECM bioinks including Bone deCelluid™, Cartilage deCelluid™, Skin deCelluid™, and Gel4Tissue^®^ [30,70,71,72]. The dECM bioink is then ready to be equipped for 3D bioprinting.

### 3.2. Considerations of dECM Bioink for 3D Bioprinting

Well-prepared dECM materials must be free of genetic matter from cells (e.g., DNA and RNA) to eliminate the risk of immunogenicity. They should nonetheless contain abundant and diverse ECM components that resemble the conditions present in their natural tissue counterparts, which is recognized as tissue specificity. Although recent evidence emphasizes that dECM materials are promising bioinks, it is important to understand that there are several critical requirements to meet before applying dECM as a bioink for 3D bioprinting, including printability, cell compatibility, mechanical properties, and remodeling capacity.

#### 3.2.1. Printability

Excellent printability refers to the capacity of a bioink to support precise 3D bioprinting of a living-cell-laden construct with pre-designed structures [73]. Achieving satisfactory printability is highly dependent on the properties of applied bioinks, bioprinter configurations, and biofabrication strategies [74,75]. The dECM bioink should possess an appropriate viscosity that enables tissue fabrication using 3D bioprinting technology, as analyzed in Section 2. Viscosity is a natural characteristic of a fluidic material, referring to the internal frictional force between adjacent layers of fluid that are in relative motion. As in other fluids, the viscosity of dECM bioink is determined by the nature of the dECM materials, such as molecular weight, molecular structure, and interactions between chains. Because different tissues contain various ECM components, the resultant tissue-specific dECM bioinks inevitably show disparate viscosities. For instance, the reported dECM bioink obtained from the porcine brain has much lower viscosity (2.1 ± 0.3 Pa, 10 mg/mL) [76] than that harvested from porcine skin (300–500 Pa, 10 mg/mL) [30], which might be due to the lack of elastic proteins (e.g., collagen and elastin) in brain tissues. Although viscosity is the nature of a material, it can be tuned by controlling temperature; in general, viscosity is positively correlated with temperature [77]. However, due to the thermal sensitivity of collagen components as well as considering cell viability, the range over which temperature can be tuned during 3D bioprinting is usually confined between 4 °C and 37 °C. In addition to manipulating their temperature, increasing the concentration of dECM bioinks could also effectively elevate their viscosities. However, dECM materials have limited water solubility. Hence, it is difficult to control viscosity freely by changing their concentration. Intractable viscosity is a common drawback of bioinks originating from natural polymers. Supplementing thick materials as additives may be an effective way to solve this problem.

In addition, a rapid and irreversible sol–gel transition behavior is another key criterion for good printability. Ideally, the liquid bioink should be immediately polymerized after being deposited on demand, forming stable solid hydrogels that retain the printed structures. Most of the currently available dECM bioinks rely on the thermal gelation of collagen components, a process of fibril formation induced by peptide bonds between collagen molecules [78]. However, this process usually requires over 10 min to accomplish [79], and thus leads to the poor printability of dECM bioinks. To overcome this limitation, various strategies have been applied to improve the performance of tissue-specific bioinks, such as the incorporation of additives/crosslinkers and the chemical modification of dECM bioinks, which can help enable other rapid polymerizations (e.g., ionic and photo-gelation) [80,81].

#### 3.2.2. Cell Viability

Achieving high cell viability in cell-laden constructs is the first objective of 3D bioprinting. To ensure that the cells remain viable during the microextrusion-based printing process, the dECM bioink must exhibit a shear-thinning behavior to reduce the generated shear stress when flowing through a narrow nozzle. Reportedly, shear-induced cell damage can be minimized when the shear stress is less than 5 kPa [82]. Thus far, all types of tissue-specific bioinks have exhibited non-Newtonian behavior and shear-relaxation properties. However, because the shear stress is determined by both the viscosity of the bioink and the size of the printing nozzles, it is important to consider the detrimental effects of shear stress before selecting the printing devices and formulating dECM bioinks. The range of suitable concentrations of currently developed bioinks that can guarantee cell viability and function is 0.6–3% [53].

Nutrient/oxygen supply and metabolic byproduct removal are essential for the survival of cells. To secure cell viability, one of the easiest ways is to physically regulate the microstructure of the biological construct, so that the necessary regulating processes can be realized in vitro. Adjusting the porosity of bioink hydrogels can efficiently control the permeability of the cell-residing microenvironment, and thus the metabolic transportations can be effectively mediated [83]. The recently developed advanced 3D bioprinting strategies have achieved high fabrication resolution, dozens of micro-meters, that can be used to reconstruct microchannels and micro-vasculatures. For example, heart and liver dECM bioinks were successfully applied to construct biomimetic tissues with microchannels and interconnected vascular networks (30 μm to 180 μm wide) using a scanningless and continuous 3D bioprinting method [84]. Such defined ultrafine structures can directly deliver nutrients and oxygens to living cells.

In addition, the polymerization process that converts bioink into a stable hydrogel is another stage that might affect cell viability. The three main methods of polymerizing bioinks are thermal crosslinking, photo-crosslinking, and ionic crosslinking. First, as previously mentioned, the gelation of the dECM bioink depends on the polymerization of collagen components. Although thermal gelation via simple incubation does not cause detrimental effects on cell viability, different concentrations of dECM bioinks might result in altered gelation kinetics, which subsequently requires a prolonged or reduced incubation period to avoid insufficient gelation or dehydration-induced cell damage. Second, for photo-crosslinking, photo-initiators are necessary to initiate free radical reactions under ultraviolet (UV) light irradiation. Although dECM materials do not support photopolymerization, it is possible to achieve this by blending photo-crosslinkable materials with dECM bioinks. For instance, one study applied riboflavin to reinforce the cardiac dECM bioink after photopolymerization under UV light [85]. However, photo-initiators are generally toxic to cells, and UV light can damage cell DNA, thereby reducing cell viability. Therefore, to ensure high cell viability, it is important to carefully consider the concentration of the photo-initiator and control UV irradiation to provide a cell-friendly polymerization process. Lastly, ionic gelation relies on the presence of ion crosslinkers in the bioink. Alginate is a commonly used bioink that can be ionically crosslinked. When this polysaccharide polymer encounters divalent cations (e.g., Ca^2+^, Ba^2+^, Mg^2+^), instant crosslinking of the carboxylate groups of the guluronate groups occurs on the polymer backbone, forming hydrogels. A hybrid bioink containing vascular dECM and alginate has been formulated to facilitate coaxial bioprinting of blood vessel constructs involving human endothelial cells [86]. The concentration of ions should be sufficiently high to fully polymerize the alginate components. However, excessively high ion concentrations may adversely cause cell death due to osmotic pressure.

#### 3.2.3. Mechanical and Compositional Stability

As the printed bioink is polymerized to form hydrogels, its role changes from a cell carrier to a scaffold that mechanically supports the printed tissue analogs and provides biological cues to promote the activities of embedded cells until they synthesize and assemble sufficient ECM instead. Therefore, appropriate mechanical strength and stable compositional disintegration are critical requirements for dECM bioinks.

First, as a scaffold, the bioink hydrogel should be sufficiently strong to resist gravity-caused structural collapse to sustain the complex 3D constructs. Moreover, because the hydrogel is composed of tightly bonded polymer chains, the 3D network absorbs additional water content from the culture medium during the incipient incubation period, leading to structural swelling. A hybrid bioink containing 3 wt% vascular dECM and 2 wt% alginate was reported to have swelled by 150.61 ± 6.37% before reaching equilibrium (10 h) [87]. Structural dilation may cause loss of resolution and a reduction in porosity. Furthermore, as the encapsulated cells attach, proliferate, and migrate, the generated force can induce severe shrinkage of the hydrogel, resulting in the dilapidation of the fabricated constructs. Hence, attention should be paid to minimizing the swelling and contraction of the hydrogel by modulating the mechanical strength of the dECM bioink. Various strategies have been proposed for 3D bioprinting of robust hydrogels. For example, a bioink consisting of alginate and poly (ethylene glycol) diacrylate was developed as an interpenetrating network via ionic–covalent bonding entanglement [88]. The printed structures could resist mechanical stress without significant plastic deformation while maintaining high cell viability (75.5 ± 11.6%) over 7 days.

In addition, upon 3D bioprinting of cell-laden constructs, dynamic ECM remodeling is an important process for successful tissue morphogenesis. In particular, the bioink hydrogel should guide the residing cells to proliferate, differentiate, aggregate, and reorganize; meanwhile, it needs to be gradually degraded and replaced by the de novo ECM produced by cells. The dECM bioinks can provide a superior microenvironment for cell-cell and cell-ECM interactions. However, achieving a tunable degradation rate of bioink hydrogels remains challenging. Rapid degradation might cause compositional instability, weakening the advantage of the dECM bioink, while retarded disintegration of the hydrogel could impede cell activities. Although it is possible to add degradation-active/inert ingredients (e.g., alginate and agarose) to manipulate the degradation rates of the bioink [89], the options remain limited considering the viability and activities of cells. Thus, for 3D bioprinting of functional tissues and organs, the degradation rate of bioink hydrogels must be prudently designed.

## 4. Applications

### 4.1. Bone

Bone is a multifunctional hard tissue that supports the mechanical actions of the body, protecting internal organs and preserving vital minerals [90]. Osseous tissue consists of four types of cells—osteoprogenitor cells, osteoblasts, osteocytes, and osteoclasts—and extracellular matrix, which is abundant in organic components, including osteocalcin, osteopontin, osteonectin, leucine-rich proteoglycan, aggrecan, HA, polysaccharide, GAGs, and type I collagen, and inorganic components, including hydroxyapatite and calcium carbonate [91,92]. As a composite material, bone exhibits excellent tensile strength and elasticity. In particular, collagen and hydroxyapatite are the major components that provide increased flexibility and stiffness [93]. In response to fractures or other defects, such as traumatic injuries and diseases, the cells orchestrate the deposition of bone matrix and maintain structural and functional stability [58]. However, if the size of the damaged tissue is over 2.5 cm, tissue regeneration stops [94]. Large segmental defects disrupt homeostatic constancy and lead to additional complications, including osteoporosis and osteopenia [95,96]. To overcome this issue, the demand for the development of tissue-engineered bone tissue substitutes has increased.

Miscellaneous biomaterials have been investigated to replace injured bone tissues [97,98]. Conventionally, prosthetic implants fabricated of titanium or poly(methyl methacrylate) have been inserted into the defect site. The grafts provide motion freedom and supportive function with superior mechanical properties; however, the integration of materials with native tissue is virtually impossible. Moreover, in young people, retransplantation surgery may be required due to bone growth. Therefore, tissue engineering of bone has attracted considerable attention as a new therapeutic solution that may overcome the current limitations [99]. Biomaterials, such as calcium phosphate ceramics, hydroxyapatite, β-tricalcium phosphate, bioactive glasses, titanium, tantalum, collagen, fibrin, alginate, silk, chitosan, and their composites have been investigated and applied in bone tissue engineering research [100,101]. However, due to the limited regenerative capacity resulting from insufficient compositional and structural similarity, integration with the surrounding native bone tissue remains a significant challenge. These issues prompted the development of bone dECM, and the material has been regarded as one of the most promising candidates for bone tissue engineering because of its distinctive biocompatibility and biofunctionality [102].

Along with the advances in 3D bioprinting technology, bone dECM has been applied as a bioink, and systemic replication of the organic and inorganic microenvironment of bone tissue has been realized based on the spatial distribution of the functional molecules [103]. Parthiban et al. functionalized bone dECM bioink using photo-crosslinkable methacrylic (BoneMA) residues [104]. BoneMA-equipped bioprintability has tunable mechanical properties which depend on the light exposure time; the realized modulus of the material was 1.56 ± 0.1 kPa. Biocompatibility was verified using encapsulated human dental pulp stem cells. Furthermore, the rapid formation of vascular networks was facilitated by angiogenic peptides residing in the bone dECM, compared with the methacrylated collagen group. Although the functionality for vascularization, which should be focused on to promote bone regeneration, was demonstrated, the mechanical strength was still weaker than that of native bone tissue (cortical bone: 10.9–34.3 GPa; cancellous bone: 0.01–1.61 GPa) [105]. Together with the need for encapsulated cells, a new strategy for physical enhancement should be sought.

**Figure 3:**
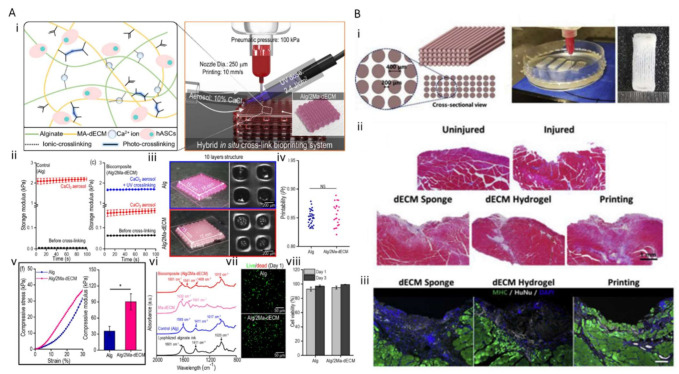
Three-dimensional bioprinting of bone and muscle. (**A**) (**i**) A 3D bioprinted bone construct using Alg/2Ma-dECM; (**ii**–**vi**) comparison of physical properties including storage modulus, printability, compressive stress, and photo-absorbability in 3D bioprinted bone constructs using alginate and Alg/2Ma-dECM; (**vi**–**viii**) comparison of biocompatibility of 3D bioprinted bone constructs using alginate and Alg/2Ma-dECM (*, *p* < 0.05); (**B**) (**i**) 3D-bioprinted skeletal muscle construct using mdECM; (**ii**,**iii**) comparison of regenerative capacity through Masson’s trichrome staining and immunostaining against MHC and HuNu. Reproduced with permission from Refs. [106,115] with slight changes [106,115].

Lee et al. developed a bone-derived dECM/alginate bioink for fabricating a 3D cell-laden mesh structure for bone tissue engineering (Figure 3A) [106]. dECM was obtained by decellularizing the bone tissue and methacrylating it to enhance the printability of the hydrogel. Furthermore, alginate was mixed with the hydrogel compound (Alg/2Ma-dECM). The printability and cell viability of the composite bioinks were optimized. A striking differentiation of human adipose stem cells was observed. Two strategies, methacrylation and mixing with alginate, were applied to the dECM bioink to enhance the mechanical properties and to fabricate the freestanding structure. The Alg/2Ma-dECM bioink was crosslinked using a CaCl_2_ aerosol and UV treatment. Although superior vasculogenic potential was presented, and the mechanical properties of bone dECM bioinks were reinforced by adding photo-crosslinkable chemicals, the compressive modulus (90.4 ± 14.9 MPa) was still weaker than that of native bone tissue. To promote osteogenic activity and develop a strategy for mechanical reinforcement, an approach to ensure the construct has vascular integrational capability with host tissue should also be considered.

### 4.2. Skeletal Muscle

Skeletal muscle is a voluntary muscle that comprises multiple bundles of fibers that performs machinery functions of the body under conscious control of the somatic nervous system [107]. In particular, the muscle has a distinctive structural feature: the sarcomere, which is the smallest functional unit of a skeletal muscle fiber, is systemically organized. Constantly contracting and expanding, highly aligned fibers constantly generate specific movements in correlation with the nervous system. In addition, the soft tissue supports a constant blood flow for nutrient exchange using motional capability [108]. Despite the remarkable capacity for regeneration, irreversible volumetric and functional loss against large external damage is unavoidable against volumetric muscle loss (VML) [109]. Defects are accompanied by functional impairment, disability, and chronic pain. Although transposition of autologous tissue is the current gold standard of clinical approach for VML, it inevitably leads to donor site morbidity [110].

To offer potential solutions, various engineering approaches for in vitro and ex vivo culturing of skeletal muscle tissue are being attempted for the regeneration of defective muscles. Studies on skeletal muscle tissue culture have been conducted for more than a century. Various novel approaches have been applied in skeletal tissue engineering. To realize an anisotropic microenvironment that is favorable for muscle alignment, micro/nanopatterning of chemicals and electric/mechanic/magnetic stimulations have been applied using various biomaterials, such as polycaprolactone (PCL)-based polymers, alginate, and fibrin, for skeletal tissue engineering [111,112]. However, despite these evolutionary advances, fully functional in vitro skeletal tissues have not yet been developed.

Among recent advances, research using 3D bioprinting technology and muscle dECM bioink has attracted attention because it has a high potential to recapitulate the skeletal tissue microenvironment in a spatiotemporal manner with more native-like multiple protein components. Choi et al. developed skeletal muscle dECM (mdECM) bioink, which preserves the complex biochemical composition including laminin, fibronectin, and collagen to recapitulate the physiological features of the muscle [113,114]. Furthermore, muscle constructs were printed using mdECM in a gelatin granule-based printing reservoir and treated with polyvinyl alcohol to overcome the low viscosity and mechanical stiffness issues of the bioink (Figure 3B) [115]. Along with the differentiation of myoblasts, the nuclei of the cells were fused, and multinucleated myotubes were formed. The matured muscle constructs were implanted into the injured tibialis anterior muscle, and the results demonstrated that mdECM facilitates rapid de novo muscle regeneration. However, because skeletal muscle tissue has highly complex nervous and vascular networks, further studies on multi-connective muscular tissue are required.

### 4.3. Cartilage

Cartilage is a major connective tissue, residing at the edges of bone tissues, that dissipates concentrated loads and promotes low-friction motions. This avascular, alymphatic, and aneural tissue is composed of approximately 80% water, ECM, and chondrocytes [100]. In particular, its distinctive mechanical features are determined by ECM components, type II collagen (the predominant macromolecule that promotes tensile strength), and GAGs (the second largest protein group that provides high compressive loads) [116,117,118]. Unfortunately, because most of the cartilage has a limited number of chondrocytes (less than 5%) and no blood vessels and nerves, the cartilage tissue is vulnerable to injury and wear over time, and recovery and regeneration of osteochondral tissue are particularly challenging compared to those of other connective tissue. Regeneration is possible only when the cells that make cartilage constantly exchange signals with the human body and create a solid tissue, but this process takes a long time, from several months to several years. Even if the damaged cartilage is cured, stiffer fibrous cartilage is formed, which limits the long-term performance of the tissue [119].

Current multifarious approaches to substitute defective cartilage tissue have been presented. The most commonly used materials are collagen, chitosan, gelatin, fibrin, polycolide, alginate, and HA [120]. Interestingly, biomaterials have been commercialized for clinical applications (e.g., MACI^®^, Chondro-Gide^®^, Maix^®^, Atelocollagen^®^, Hyalograft^®^ C, and Bio-Seed^®^-C) [121]. However, the need for the development of materials with the same compositional diversity as the actual tissues has been repeatedly raised. Furthermore, cartilage has a compartmentalized ultrastructure. Therefore, it is essential to introduce a technology with a high degree of freedom that can reproduce its sophisticated structural complexity [122].

**Figure 4:**
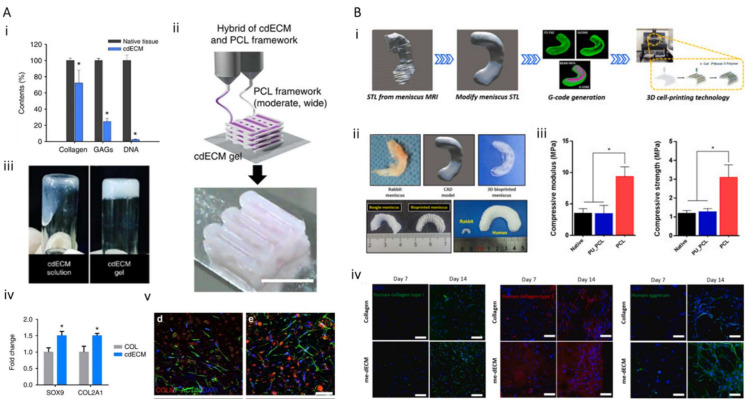
Three-dimensional bioprinting of cartilage. (**A**) (**i**) Biochemical analysis of cdECM bioink; (**ii**) 3D bioprinted cartilage construct; (**iii**) sol-gel transition of cdECM; (**iv**,**v**) relative mRNA expression of cartilage-specific ECM (scale: 50 μm; *, *p* < 0.05). (**B**) (**i**,**ii**) 3D bioprinted implantable meniscus graft; (**iii**) comparison of the compressive modulus and strength of 3D bioprinted meniscus; (**iv**) comparison of immunostaining of type I collagen, type II collagen, and aggrecan in collagen and me-dECM groups on day 7 (*, *p* < 0.05). Reproduced with permission from Refs. [71,124] with slight changes [71,124].

These demands naturally result in the application of cartilage tissue-specific dECM bioink and 3D printing technology for cartilage tissue engineering. Pati et al. developed cartilage dECM (cdECM) to reconstruct cartilage tissue in vitro and observe the spatial patterns and tissue-specific gene expression level along with tissue maturation (Figure 4A) [71]. Type II collagen and GAGs were preserved, and dsDNA segments were eliminated (11 ± 1 ng/mg dECM). Human inferior turbinate tissue-derived mesenchymal stromal cells (hTMSCs) were encapsulated within cdECM and printed on a 3D bioprinted PCL framework. Although the mechanical behavior and cellular functionality depending on maturation were not fully estimated, this suggests the potential of the use of cartilage dECM bioink for cartilage tissue regeneration. Zhang et al. developed a crosslinker-free bioink by combining a cartilage extracellular matrix with silk fibroin (SF-dECM bioinks) to promote chondrogenic differentiation in a cartilage-specific microenvironment. The mechanical strength and shape tenability of the bioink were enhanced; this strategy can be regarded as a promising approach for cartilage regeneration [123]. While proliferation and chondrogenic differentiation of mesenchymal stem cells were promoted, their mechanical properties were not comparable to those of native cartilage tissue. Chae et al. developed meniscus, half-moon-shaped fibrocartilage-derived dECM bioink and fabricated implantable cell-printed meniscus constructs using a polymer mixture of polyurethane, PCL, and cell-laden me-dECM bioink (Figure 4B) [124]. The constructs not only possessed favorable biocompatibility and microenvironment promoting neofibrocartilage formation through fibrochondrogenic differentiation of the encapsulated stem cells, but also exhibited tensile properties to resist external pressure. However, cartilage replacement with full functionality and spatial complexity has not yet been realized, and significant technical progress is required.

### 4.4. Tendon

A tendon is a tough bank composed of dense fibrous connective tissue. It connects the skeletal muscle to the bone and transfers tension during body motion. The cells residing in the tendon are mainly tenocytes, a type of specialized fibroblasts. Tenocytes secrete abundant densely packed collagen fibers that are laterally organized in a parallel fashion to form fascicles. Numerous fascicles are bound together (endomysium), which are enclosed by a sheath of dense connective tissue (epimysium) and filled with loose connective tissues (perimysium) to produce mature tendons. The ECM of tendon tissue contains multiple types of collagens (e.g., type I, III, and IV) and complex non-collagen components (e.g., cartilage oligomeric matrix protein, elastin, proteoglycans). The assembly of these ECM components, as well as their organization, results in high tendon tissue strength. The ultimate tensile strength of the human tendon ranges from 50 to 150 MPa [125].

Although minor ruptures of tendon tissue can self-heal through the response of local cells toward the injuries, greater damage requires surgical interference such as suture surgery or graft implantation to repair or regenerate the tendon. However, autograft implantation has limited regenerative ability to resume the robustness of tendon tissue. Thus, advanced strategies have been suggested to promote tendon regeneration, such as cell therapy [126,127], novel biomaterials [128], and refined scaffold designs [129]. However, the construction of an artificial tendon with strong mechanical properties analogous to that of native tissue has yet to be achieved. Herein, we review the progress and contributions of 3D bioprinting and tendon dECM bioink to the advent of reliable tendon equivalents.

As an anisotropic tissue, the high tensile strength of the tendon is derived from the unidirectional orientation of its ECM components. Inspired by this anatomy, one study fabricated a scaffold with highly oriented PCL fibers along the uniaxial direction using an electrohydrodynamic jet printing method [130]. This ECM-mimicking distribution effectively guided the alignment of human tenocytes upon cell seeding and seven-day culture. However, this desirable cell and fiber alignment still failed to attain a sufficient tensile strength comparable to that of native tendon tissue (4.5 ± 0.6 MPa vs. >50 MPa). This might be attributed to the weak mechanical properties of PCL and limited activities of the involved tenocytes, which hamper the maturation of tendon tissue. Another study attempted to overcome these limitations by developing a hybrid scaffold that embeds a 3D printed acrylonitrile butadiene styrene scaffold into a collagen-GAG sponge [131]. While the acrylonitrile butadiene styrene core offers mechanical support, the bioactive collagen-GAG shell can promote cell migration and thus accelerate tissue regeneration. Nonetheless, its ultimate strength (0.91 ± 0.063 MPa) is still lower than that of native tissue.

In addition to seeding cells onto a processed scaffold, 3D bioprinting is a more advisable technique for constructing analogous tendons because of its ability to directly orchestrate cells and biomaterials. In a recent study, a collagen-fibrin hybrid bioink containing human adipose-derived mesenchymal stem cells (hAMSCs) was bioprinted into a polylactic-co-glycolic acid framework fabricated previously [132]. The composite tendon substitute showed an elastic stiffness of 60 N/mm (elastic stiffness of native human Achilles tendon: 201.8 ± 5.9 N/mm) and an ultimate failure force of 17 N. In addition, the embedded hASMCs were differentiated into tenocytes upon cultivation in a tendon differentiation medium.

**Figure 5:**
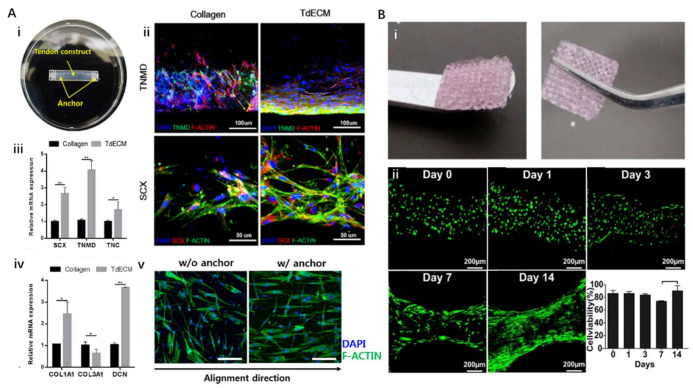
Three-dimensional bioprinting of tendons and ligaments. (**A**). (**i**) A 3D bioprinted tendon construct; (**ii**) comparison of immunofluorescence staining against TNMD and SCX in 3D bioprinted tendon constructs using collagen and TdECM on day 7; (**iii**,**iv**) relative mRNA expression of tendon-specific and ECM genes in tendon constructs in collagen and TdECM groups at day 7; (**v**) confocal fluorescence images of tendon constructs at culture day 14 without and with anchor (scale: 50 μm). (**B**). (**i**) Thermally crosslinked 3D bioprinted GelMA hydrogel scaffolds; (**ii**) viability of periodontal ligament stem cells embedded in the 3D bioprinted constructs during 14 days. (*, *p* < 0.05, **, *p* < 0.05). Reproduced with permission from Refs. [133,138] with slight changes [133,138].

Although few studies have focused on 3D bioprinting of tendon tissue, a recently published study has demonstrated the potential of the tendon dECM (TdECM) bioink (Figure 5A) [133]. A TdECM bioink formulated from decellularized porcine Achilles tendon was used to encapsulate human bone marrow-derived mesenchymal stem cells (hBMMSCs). The cell-laden bioink was 3D bioprinted to build a designed tendon construct that could induce tensile force stimulation upon hydrogel contraction. The embedded cells remained highly viable (>98%) over 14 days. Moreover, compared with the bioink composed of type I collagen, the TdECM bioink significantly promoted the differentiation of hBMMSCs toward the tenocyte lineage, with the upregulation of tenascin-C, tenomodulin, scleraxis, and decorin, which are specific markers of tenocytes and tendon ECMs. In addition, the cells embedded in TdECM showed highly aligned fiber orientation during maturation, whereas a randomly aligned orientation was observed in the collagen-based bioink group. However, the mechanical properties of individual tendon tissues have not been reported. Nevertheless, these findings demonstrate that the developed TdECM bioink could improve tendon-specific differentiation and augment tendon-like tissue formation.

### 4.5. Ligament

The ligament is compositionally and structurally comparable to the tendon, which is also a fibrous connective tissue formed by oriented, hierarchized fibers mainly composed of collagen. Thus, ligaments show anisotropic properties similar to tendons, such as unidirectionally strong ultimate tensile strength (50–150 MPa) [134]. In addition, the cells residing in the ligament, called fibrocytes, have an identical role to the tenocytes in tendon tissue, which are responsible for composition and repair of the ECM components. The difference between tendons and ligaments lies in their locations and functions. While tendons are found adjacent to each skeletal muscle for transferring force from muscle to bone, the ligaments are located around joints attaching bone to bone to stabilize the joints. In addition, the cells residing in the ligament are mostly fibrocytes, which are different from the tenocytes in tendon tissue.

Although ligaments are strong, they can be overstretched or even torn when people engage in excessive activities, resulting in varied grades of tissue damage, including sprains (no joint laxity), partial ruptures (moderate joint laxity), and complete ruptures. Injury to the anterior cruciate ligament is one of the most common ligament injuries. Severe anterior cruciate ligament injury can lead to knee joint instability and thus usually requires surgery or reconstructive grafting using autologous tendon/ligament, allografts, and artificial ligament substitutes [135]. Although these strategies have demonstrated the feasibility of ligament repair, adverse effects, such as graft rejection, donor site morbidity, and insufficient osteointegration have limited their clinical applications. These limitations have provoked extensive interest in the development of tissue-engineered ligaments that integrate cells, growth factors, and biomimetically designed scaffolds to upregulate ligament regeneration [136].

Recently, researchers attempted to create ligament grafts using 3D bioprinting technology. For instance, one team seeded periodontal ligament stem cells onto the 3D printed PCL scaffold [137]. They studied the influence of the thickness and porosity of the scaffold on the proliferation rate and mineralization function of periodontal ligament stem cells and demonstrated the feasibility of integrating ligament cells with a 3D structure. Moreover, direct 3D bioprinting of primary periodontal ligament cells encapsulated in the methacrylated gelatin (GelMA) bioink has been reported (Figure 5B) [138]. However, these pioneering studies merely focused on the successful fabrication of 3D ligament constructs with high cell viability, rather than generating functionally and mechanically biomimetic ligament grafts. In addition, no ligament-specific dECM bioink has been reported as yet, which also leaves a vacancy for future efforts.

### 4.6. Vascularized/Innervated Musculoskeletal Tissues

Vasculatures and neural networks are important circulatory and sensory systems in musculoskeletal tissues. Blood vessels provide essential metabolic nutrition and oxygen to secure the viability of tissues. It has been demonstrated that cells residing over a 200 μm distance from a blood vessel would be dead due to the resulting hypoxic conditions [139]. Hence, for any tissue-engineered construct, the presence of vasculature is necessary. The neuromuscular junction (NMJ) is responsible for the most important relationship between the nervous system and musculoskeletal tissues. The nervous system transfers communicating messages from the brain to skeletal muscles to enable body motions; however, extensive injury of musculoskeletal tissue might impinge this association. A study focusing on rat tibialis anterior VML revealed that 20% mass loss of skeletal muscle not only yielded muscular strength deficits ranging from 45% to 90%, but also made from 57% to 79% of motor neurons undergo significant axotomy and lost interactions with the TA muscle [115]. In addition, neural disorders can lead to musculoskeletal diseases. For example, amyotrophic lateral sclerosis, induced by the degeneration and death of motor neurons, can lead to skeletal muscle denervation, atrophy, and, most often, death of the patient within five years of diagnosis. Hence, to upgrade the regenerative outcomes, it is necessary to incorporate these critical elements to construct vascularized/innervated musculoskeletal tissues.

#### 4.6.1. Three-Dimensional bioprinting of Vascularized Musculoskeletal Tissue

The mechanism of neovascularization can be roughly divided into angiogenesis and vasculogenesis. Angiogenesis is the growth of new capillaries from pre-existing blood vessels, while vasculogenesis refers to the formation of a primitive vascular network from endothelial primary or progenitor cells. Accordingly, two prevailing strategies have been established for 3D bioprinting of vascularized tissue constructs: the incorporation of angiogenic factors and pre-vascularization.

**Figure 6:**
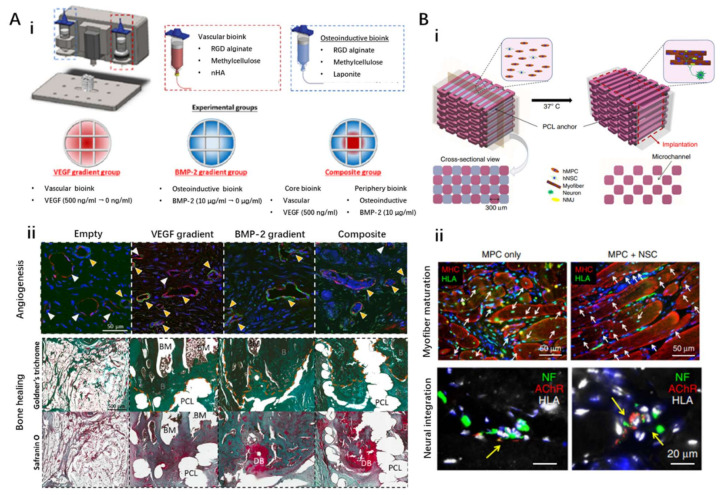
Three-dimensional bioprinting of vascularized and innervated musculoskeletal tissue. (**A**). (**i**) Schematic diagram of 3D bioprinting with specific bioinks and designed experimental groups; (**ii**) spatially localized VEGF and BMP-2 leads to improved angio-genesis and bone healing effects. (**B**). (**i**) Schematic diagram of the 3D bioprinted innervated skeletal muscle construct: the cell-laden bioink containing human muscle progenitor cells (hMPCs) and/or hNSCs, the acellular sacrificing bio-ink, and the supporting PCL pillar were deposited by multi-dispensing modules, and the microchannels in the con-structs were created upon the removal of the sacrificial pattern; (**ii**) comparison of myofiber maturation and neural integration performances under different experimental conditions (MPC only vs. MPC + NSC). Reproduced with permission from Refs. [140,143] with slight changes [140,143].

The first strategy attempts to spatially involve growth factors (e.g., vascular platelet-derived growth factor, vascular endothelial growth factor (VEGF), fibroblast growth factors) that can induce the ingrowth of host blood vessels into the fabricated construct, realizing in vivo angiogenesis. Thus, numerous studies have successfully achieved vascularized bone and muscle tissue. For instance, one study utilized 3D bioprinting technology to fabricate a biomimetic vascularized bone construct with regional immobilization of BMP-2 and VEGF. The gradient of growth factors loaded in the engineered constructs exhibited a superior ability to enhance microcapillary formation (Figure 6A) [140]. However, biomolecules usually have a short half-life and are rapidly eliminated in vivo, which might lead to insufficient dosage for the generation of viable vascular networks that support implanted grafts. Although encapsulating growth factors into polymeric particles and microspheres can achieve prolonged release kinetics, maintaining the gradient angiogenic signals remains challenging. The application of dECM bioink might be an alternative to such an elaborate design. Gao et al. developed a vascular dECM bioink from porcine aorta tissue for treating ischemic diseases. In particular, a tubular construct containing human endothelial progenitor cells and microspheres loaded with atorvastatin were successfully fabricated using coaxial 3D bioprinting technology [141]. After implantation into the ischemic limb of mice for 28 days, such a cell/drug co-laden vessel showed remarkable neo-vessel formation and limb salvage, demonstrating the potential of VdECM to promote neovascularization. Despite these remarkable achievements, angiogenesis-based vascularization is highly dependent on complex in vivo microenvironments, cellular status, construct design, and release profile of growth factors, which inevitably make the strategy difficult to control.

In addition, coaxial 3D bioprinting of the VdECM bioink could also contribute to the prevascularization of tissue constructs. Using this technique, Choi et al. combined the advantages of MdECM and VdECM to directly bioprint a core/shell filament that spatially carries human umbilical vein endothelial cells and human skeletal muscle cells that emulate the hierarchical architecture of vascularized muscle fibers [115]. Subsequently, multiple prevascularized muscles were fabricated together to scale-up a large-volume muscular construct for treating VML created in rat models, which extensively improved muscle fiber formation, neovascularization, and recovery of muscle contractile force. Apart from the direct printing of vascular constructs and positioning endothelial cells, 3D bioprinting can also realize the prevascularization of tissue constructs by creating hollow channels in bulk cell-laden hydrogels. Kolesky et al. applied cell-laden GelMA to embed a template of vasculature 3D printed using Pluronic F-127 [142]. Upon polymerization of GelMA, Pluronic F-127 was liquefied to facilitate the evacuation of the template. After seeding by endothelial cells, the resultant interconnected microchannels in the matrix resembled the native vasculature. Although engineering hollow channels can be controlled to effectively configure the pattern and design of vasculatures, the shrinkage of the hosting matrix caused by the activity of embedded cells might induce structural deformation, leading to destructive dilapidation and channel blockage. Therefore, careful design of the bioink formulation and evaluation of mechanical and rheological properties are important when this strategy is considered.

#### 4.6.2. Three-Dimensional bioprinting of Innervated Musculoskeletal Tissue

Compared with vascularization, research regarding tissue innervation began relatively late, and its progress is still in its infancy. Denervated skeletal muscles lose their contractility and suffer from muscle atrophy. Tissue-engineered skeletal muscle constructs consisting of living muscle cells are similar to denervated native counterparts, which require rapid integration with the host nervous system. Otherwise, muscle atrophy and functional recovery failure occur. The primary methods to encourage innervation of regenerating muscle after VML treatment are neurotization and rehabilitative exercise. However, because both methods result in only moderate success, advanced constructs with mature neural tissues must be developed to improve the therapeutic effects for VML or to better understand the development and maturation of NMJs.

The 3D bioprinting technique is advantageous in precisely integrating multiple cells and biomolecules within a complex entity and thus could promise effective incorporation of nerves with muscle constructs. Kim et al. investigated the feasibility of establishing a neural cell-integrated human skeletal muscle equivalent using a 3D bioprinting technique (Figure 6B) [143]. This construct was fabricated by 3D bioprinting of a hybrid bioink (HA/fibrinogen/gelatin/glycerol) containing HMPCs and/or human neural stem cells (HNSCs) onto a PCL anchor fabricated previously. To avoid hypoxia-induced cell death, fugitive Pluronic F-127 was utilized to fabricate the embedded channels. The involvement of neural cells in the skeletal muscle construct promoted myofiber generation, long-term survival, and NMJ formation in vitro. Moreover, the integration of HNSCs facilitated rapid innervation, restoration, maturation, and functionalization of muscle tissue in a rodent model of muscle defect injury, suggesting that the neural-skeletal muscle construct can be rapidly integrated with the host neural network, leading to accelerated muscle regeneration. In addition, the coaxial 3D bioprinting technique has also been utilized to co-print primary myoblasts and NSC-34 cells (a motor neuron cell line) [144]. Such a core/shell structure mimics the muscle/neuron physiological system by bringing differentiating motor neuron-like cells in proximity with skeletal myoblasts. Although the formation of mature NMJs has not been reported, the improved differentiation of NSC-34 cells in proximity to skeletal myofibers in the coaxial scaffolds is in accordance with the presence of skeletal myoblasts, which are known to enhance the development of neuromuscular junctions.

### 4.7. Interfaces of Musculoskeletal Tissues

Musculoskeletal interfaces (MI), the transition sites of dissimilar musculoskeletal tissues, are vulnerable to wear and tear from injury because of their anisotropic structural and mechanical properties from soft to hard tissues. Traditionally, orthopedic interfaces have been accessed by autograft or allograft tissue transplantation, but they are accompanied by problems such as loss of the donor site, disease infection, and immune response. To overcome these limitations, advanced tissue engineering approaches using 3D bioprinting technology have recently gained attention because they allow the organization of a complex arrangement of functional factors. However, bioprinting MI is still in the initial stages of research because the understanding of the biomechanical features of orthopedic interfaces is limited [23]. Focusing on the best bioink formulation to recapitulate native mechanical properties, the construction of hierarchical organization and cellular functionalities to external stimuli has been investigated.

Bone-cartilage interfaces in large joints, such as the knee and hip joints, are particularly susceptible to injuries. Due to the poor regenerative capability of osteochondral tissues, the defects are fatal. Turner et al. reported the regenerative potential of the small intestine submucosa-derived extracellular matrix to cartilage and bone formation [145]. The scaffolds made up of the ECM were implanted to the resected site among the muscle, tendon, and bone of 16 dogs. In addition to the formation of dense collagenous tissue, ossification of the lesions was also observed. Osteogenic and chondrogenic differentiation of MSCs was promoted. This demonstrates the potential of dECM for bone-cartilage interface repair. Yang et al. printed an osteochondral graft [146]. Using cartilage dECM/alginate bioink and alginate/hydroxyapatite bioink, a multilayered structure of chondral and osseous tissue could be reproduced. The biphasic graft showed hierarchical cellular deposition and higher structural integrity without collapse. The potential of 3D bioprinting technology and tissue-derived dECM bioink was verified by creating a heterogeneous osteochondral graft.

Bone-tendon/ligament interfaces play an important role in transferring loads from the muscle to the bone, thereby maintaining joint stability. However, due to its limited healing capacity, the regeneration of a torn or injured tendon-bone junction is regarded as a tremendous clinical challenge. Although metallic and polymeric grafts have been invented, reproducing the gradient of tendon-bone remains complicated [147,148,149]. Therefore, bone-tendon interfaces with spatially organized scaffolds equipped with drugs, genes, and cells with spatial organization must be engineered [150]. Recently, based on the flexibility of the 3D bioprinting technique, a spatially graded tendon-bone interface (TBI) patch was constructed using hBMMSC-laden TdECM and bone dECM (BdECM) bioinks in subsequent experiments [133]. Such a fibrocartilaginous construct significantly accelerates and promotes TBI healing in a rat chronic tear model, which demonstrates the regenerative potential of a tissue-specific dECM bioink-based approach. The fibrocartilage part at the meeting point of the tendon and bone tissue was constructed considering the complex hierarchical structure, which demonstrates the translational potential for clinical adaptation.

The muscle-tendon interface has vital physiological functions. Although there are limited studies related to the myotendinous junction in applied 3D bioprinting technology, gradients of chemical and physical cues that affect cell fate including proliferation, differentiation, and migration must be created to engineer native-like heterogeneous muscle-tendon interfaces. By controlling the pore size, material stiffness, and concentration of growth factors, spatially diverse deposition can be achieved [151].

## 5. Conclusions and Perspectives

Musculoskeletal tissue-derived dECM has shown outstanding potential for tissue engineering applications, resulting in enormous progress. Based on the compositional similarity of the biocompatible material with native tissue, specialized functions along with tissue-specific lineages can be reproduced. Furthermore, the dECM possesses versatility that can be amalgamated with 3D bioprinting technology, which allows spatial patterning to attain structural integrity of the tissue constructs. Therefore, enhanced regenerative capabilities both in vivo and in vitro have been accomplished in many studies.

Despite the biochemical resemblance and complexity of musculoskeletal-tissue–derived dECM bioinks, there are limitations that must be overcome [152]. Usually, the innate and adaptive immune responses of the donor tissue to dECM are overlooked. To minimize adverse immune reactions against the inevitable disruption of ECM ultrastructure and conformation of secondary analogs, critical analysis and optimization methods of various decellularization protocols are required. In addition to eliminating well-known antigens in dECM, such as dsDNA and GAL epitopes, understanding the interactions between ECM and immune cells, including macrophages and T-cells, is required for material approval for clinical application.

The demands on sophisticated nanostructure hierarchy of each component that make up musculoskeletal tissue have been recognized; the spatial distribution of the molecules of different sizes, shapes, folding, and assembly determine the physiological and mechanical characteristics of the tissue constructs [153]. For instance, bone displays unique physical performance. The functionality results from the hierarchical structure of hydroxyapatite crystals that exist in various lengths, from nanometers to centimeters scale, comprising up to about 60% of bone. Organizing the fundamental building blocks from the nanoscale to a large scale with precise structural alignment and sustaining the nanostructure of the components is still restricted due to technological limitations [154]. Therefore, in terms of material engineering, merging the development of nanotechnology and supramolecular chemistry is essential.

The poor mechanical strength of dECM bioinks compared with native tissues is another restriction. The decellularization process to obtain the tissue-specific functional material and the solubilization process to secure printability of a bioink inevitably leads to the loss of various components and destruction of structural stability that affect the specific physical properties such as load-bearing capabilities [102,155]. Moreover, the behavior of cells is influenced by the mechanical properties of the incorporated substrates. Not surprisingly, if physical cues, including stiffness, topography, and ligand presentation, are changed, diseases develop. While the processes for preserving biochemical components and solubilizing the dECM are focused on during decellularization, transient elastography and topography have been regarded as unavoidable processes [156]. To strengthen the weak physical properties of dECM bioinks, multiple approaches such as integrating with polymer scaffolds and incorporating into injectable dECM bioinks other synthetic and cross-linkable molecules such as polyethylene glycol diacrylate (PEGDA), vitamin B2, methacrylate, and Ru/SPS have been tried [81,85,157]. Furthermore, because the bioink has high mixability and modifiability through covalent bonds, hydrophobic and hydrophilic interactions, and electrostatic forces, cells, drugs, and growth factors can be introduced into dECM bioinks, which is also effective in recapitulating the heterogeneous microenvironment.

The musculoskeletal tissue contains soft-to-hard interface, such as ligament-to-bone, tendon-to-bone, and cartilage-to-bone. Engineering such interface tissues is more complicated than constructing a single specific tissue. It requires a combination of specialized biomaterials with spatially orchestrated compositions, structures, cell types, and biomolecules. Therefore, the conventional monophasic or composite bioinks do not reproduce the biological and physical organization of the interfaces between different tissues [158]. Hence, advanced biomaterial designs and fabrication strategies are necessary to develop biological constructs with biomimetic gradient features that can encourage the differentiation of multiple cell phenotypes and subsequent interface tissue development.

Finally, the insufficient resolution of 3D printing for reproducing spatiotemporal complexity should also be improved. The resolution depends on the technical specifications of the bioprinter type and physicochemical features of the bioink [159]. Therefore, optimization of the governing parameters, such as nozzle shape and size, printing head velocity, and bioink viscosity, can improve the resolution. Even pore size, pore interconnection, and proper surface chemistry can be controlled.

In conclusion, the explosive growth of musculoskeletal tissue engineering using 3D bioprinting technology and dECM bioink demands further studies to understand the biochemical and physical features of the musculoskeletal system, optimization of the decellularization protocol, and refinement of 3D bioprinting technology to provide proper in vivo and in vitro platforms. Through this systemic progression, these platforms can be used to identify novel therapeutic medications for disease manifestations. A multidisciplinary optimal design based on cell biology, material science, physics, and mechanical engineering is essential to overcome the recent challenges and accomplish commercialization for clinical applications.

## Figures and Tables

**Table 1:** Comparison of 3D cell-printing techniques.

	Inkjet	Extrusion	Laser
Printer cost	Low	Medium	High
Bioink viscosities	3.5–12 mPa·s	30 mPa·s to >6 × $10^{7}$ mPa·s	1–300 mPa·s
Dispensing speed	Fast (1–250,000 droplets/s)	Slow (10 μm–50 mm/s)	Medium–Fast (200–1600 mm/s)
Preparation time	Low	Medium	High
Resolution	2 μm or single cell	100 μm	~20 μm
Cell viability	>85%	40–80%	>95%
Cell densities	Low (<$10^{6}$ cells/mL)	High (cell spheroids)	Medium (10^8^ cells/mL)

**Table 2:** Comparison of properties of bioinks for 3D bioprinting technique.

Source	Bioink Type	Mechanical Property	Cytocompatibility	Printability
Natural polymer	Collagen	Weak, <1 KPa elastic moduli	Cell favorable	Poor printability
	Gelatin	Weak and unstable	Cell favorable	350–450 μm
	Fibrin	Weak, ~50 Pa	Cell favorable	Poor printability
	Silk fibroin	Strong, ~25 KPa tensile strength	Non-cytotoxic, Weak cell adhesive	280–320 μm
	Hyaluronic acid	—	Cell favorable	Poor printability
	Alginate	Tunable, varied with molecular weight and Ca^2+^ contents	Non-cytotoxic, Weak cell adhesive	Poor printability
	Agarose	Fragile, 3–15 KPa compressive strength	Non-cytotoxic, Weak cell adhesive	>500 μm
	Chitosan	—	Non-cytotoxic, Support cell adhesion	Poor printability
Synthetic polymer	Poly(ethylene glycol)	Tunable	Non-cytotoxic, Support cell adhesion	>200 μm
	Pluronic F127	Soft and weak	Cytotoxic	~150 μm

## Data Availability

Not applicable.
